# Relieving your stress: PGPB associated with Andean xerophytic plants are most abundant and active on the most extreme slopes

**DOI:** 10.3389/fmicb.2022.1062414

**Published:** 2023-01-18

**Authors:** Carla Aguilera-Torres, Gustavo Riveros, Loreto V. Morales, Angela Sierra-Almeida, Mauricio Schoebitz, Rodrigo Hasbún

**Affiliations:** ^1^Grupo de Ecofisiología Térmica, Facultad de Ciencias Naturales y Oceanográficas, Departamento de Botánica, Universidad de Concepción, Concepción, Chile; ^2^Cape Horn International Center (CHIC), Puerto Williams, Chile; ^3^Rizoma, Centro de Estudios Agroecológicos y Botánicos, Valparaíso, Chile; ^4^Laboratorio de Microbiología de Suelos, Departamento de Suelos y Recursos Naturales, Facultad de Agronomía, Universidad de Concepción, Concepción, Chile; ^5^Laboratorio de Biopelículas y Microbiología Ambiental, Centro de Biotecnología, Universidad de Concepción, Concepción, Chile; ^6^Laboratorio de Epigenética Vegetal, Facultad de Ciencias Forestales, Departamento de Silvicultura, Universidad de Concepción, Concepción, Chile

**Keywords:** alpine, ACC deaminase, extreme temperatures, IAA, nitrogenase, soil bacteria, xerophytic plants

## Abstract

**Introduction:**

Plants interact with plant growth-promoting bacteria (PGPB), especially under stress condition in natural and agricultural systems. Although a potentially beneficial microbiome has been found associated to plants from alpine systems, this plant- PGPB interaction has been scarcely studied. Nevados de Chillán Complex hold one of the southernmost xerophytic formations in Chile. Plant species living there have to cope with drought and extreme temperatures during the growing season period, microclimatic conditions that become harsher on equatorial than polar slopes, and where the interaction with PGPB could be key for plant survival. Our goal was to study the abundance and activity of different PGPB associated to two abundant plant species of Andean xerophytic formations on contrasting slopes.

**Methods:**

Twenty individuals of *Berberis empetrifolia* and *Azorella prolifera* shrubs were selected growing on a north and south slope nearby Las Fumarolas, at 2,050 m elevation. On each slope, microclimate based on temperature and moisture conditions were monitored throughout the growing period (oct. – apr.). Chemical properties of the soil under plant species canopies were also characterized. Bacterial abundance was measured as Log CFU g^−1^ from soil samples collected from each individual and slope. Then, the most abundant bacterial colonies were selected, and different hormonal (indoleacetic acid) and enzymatic (nitrogenase, phosphatase, ACC-deaminase) mechanisms that promote plant growth were assessed and measured.

**Results and Discussion:**

Extreme temperatures were observed in the north facing slope, recording the hottest days (41 vs. 36°C) and coldest nights (−9.9 vs. 6.6°C). Moreover, air and soil moisture were lower on north than on south slope, especially late in the growing season. We found that bacterial abundance was higher in soils on north than on south slope but only under *B. empetrifolia* canopy. Moreover, the activity of plant growth-promoting mechanisms varied between slopes, being on average higher on north than on south slope, but with plant species-dependent trends. Our work showed how the environmental heterogeneity at microscale in alpine systems (slope and plant species identity) underlies variations in the abundance and plant growth promoting activity of the microorganisms present under the plant canopy of the Andean xerophytic formations and highlight the importance of PGPB from harsh systems as biotechnological tools for restoration.

## Introduction

Plant growth-promoting bacteria (hereafter PGPB) are a diverse group of microorganisms beneficial to free-living or endophytic plants and can inhabit different plant compartments (de Souza et al., 2015; Afzal et al., 2017). Different bacterial traits promote plant growth directly or indirectly through mechanisms that have been widely documented, especially in soil and plant rhizosphere bacteria (Gamalero and Glick, 2011; Ramakrishna et al., 2019). These mechanisms mainly include enzymatic activities, such as urease, phosphatases, β-glucosidase, catalase, and the production of bacterial phytohormones that promote plant growth (Glick, 2012). Among the most studied mechanisms are nitrogen fixation, phosphate solubilization, phytohormone production, siderophores, and enzymes activity such as ACC deaminase that promotes resistance to different stress factors (e.g., Drought, Ashry et al., 2022; salinity, Kumar et al., 2020; trace metals, Kong and Glick, 2017). For example, the bacterial enzyme nitrogenase catalyzes molecular nitrogen to ammonia, which is absorbed by the plants increasing crop yields (Franche et al., 2009). Likewise, bacteria with ACC deaminase enzyme activity can degrade 1-aminocyclopropane-1-carboxylate (ACC), an ethylene precursor, producing ammonia and α-ketobutyrate. The decrease in ethylene levels allows the plant to survive under stressful conditions (e.g., drought and salinity; Glick et al., 2007; Orozco-Mosqueda et al., 2021). Another interesting mechanism is phosphate solubilization by bacterial enzymes such as phosphatase, phytase, and C-P lyase, that solubilize organic phosphate into inorganic phosphate, which could be absorbed and used for plant metabolism (Rawat et al., 2021). Moreover, bacterial phytohormones such as indole acetic acid (IAA) stimulate plant cell division, enhancing growth in roots and aerial structures (Duca and Glick, 2020). All these bacterial mechanisms that promote plant growth depend on environmental factors (Olanrewaju et al., 2017).

The growth-promoting activity of soil PGPB is influenced by abiotic and biotic factors. For example, nitrogen fixation is favored by increasing phosphorus and carbon availability but decreases in the presence of trace metals (Liengen, 1999; Wakelin et al., 2010). Regarding phosphate solubilization, Mujahid et al. (2015) have reported that phosphate-solubilizing PGPB increase their halo in media with carbon and nitrogen sources. While Alemneh et al. (2022) correlated soil chemical properties and phosphate solubilization of PGPB, and determined that these did not benefit from soil carbon and nitrogen. We believe that the high availability of carbon and nitrogen in growing media, such as those observed by Mujahid et al. (2015) generate opposite trends in phosphate solubilization when compared to the availability of these nutrients in the soil. Moreover, the production of IAA correlates positively with the presence of heavy metals, and negatively with the availability of sugars, nitrogen, and phosphorus in the soil (Ahmad et al., 2005; Mendoza-Hernández et al., 2016; Alemneh et al., 2022). Finally, ACC-deaminase activity of several bacteria genus increases to deaminate ACC in the presence of copper, arsenic, and lead (Mendoza-Hernández et al., 2016). Alternatively, plant root exudates compound rich in sugars, which favor microbial metabolism, increasing growth-promoting activity and plant-PGPB interactions (Bais et al., 2006). In turn, root exudates releasing is also influenced by abiotic conditions to which plants and PGPB are exposed, such as soil moisture, nutrient availability, and temperature (Vives-Peris et al., 2018; Barbosa et al., 2020). For instance, the interaction between the coastal halophyte plant *Limonium sinense* with habitat-adapted, endophytic bacteria of the genus *Bacillus* favoring plant survival under salt stress and is mediated by root exudates (Xiong et al., 2020). Soil temperature and moisture have also been defined as drivers of bacterial growth-promoting activity (Rousk et al., 2018). The ideal temperature for phosphate solubilization is below 25°C (Mujahid et al., 2015), while higher temperatures coupled with higher soil acidity increase IAA production (Kanu and Dakora, 2009; Alemneh et al., 2022). Soil moisture, in turn, has been defined as an indispensable resource for the activity of nitrogen-fixing bacteria in cold climates such as the Arctic tundra (Rousk et al., 2018). Therefore, the abundance and activity of PGPB will depend on their environmental requirements and availability of resources such as those required by the plants with which they interact.

The benefits of PGPBs have been reported mainly in agricultural systems due to the global demand for food and cultivable land (Bhattacharyya and Jha, 2012; Ramakrishna et al., 2019). In contrast, the abundance and activity of PGPBs in natural systems have been scarcely explored, despite its great potential and novelty (Pérez-Jaramillo et al., 2018; Leontidou et al., 2020). For example, PGPBs associated to wild (or native plants) species have been reported in harsh environments, such as desert (Fuentes et al., 2020; Maldonado et al., 2022), alpine (Sezen et al., 2016; Adamczyk et al., 2019; Wang et al., 2020), and saline environments (Rueda-Puente et al., 2019). Additionally, some studies have explored the presence of PGPBs adapted to environments with extreme conditions, such as salinity, (e.g., PGPBs studied from the soils of Lonar Lake in India, Hingole and Pathak, 2016), drought (e.g., PGPBs from different desert regions of Egypt, Ashry et al., 2022), or low temperatures (e.g., isolated PGPBs from the Himalayas, Yadav et al., 2015). Undoubtedly, exploring “harsh” systems for different life forms offers a space to find microorganisms with great potential for plant production and restoration, due to their ability to cope with extreme conditions and to promote plant growth and survival.

Alpine systems represent a natural laboratory to explore the presence and activity of PGPBs because of their harsh climatic conditions for life (Körner, 2004; Li et al., 2020; Rahman et al., 2020). In alpine habitats, temperature, radiation, exposure, and precipitation vary with season, altitude, and exposure of slopes (Körner and Ohsawa, 2005), producing a spatial heterogeneity of environmental conditions (Siles and Margesin, 2016; Körner, 2021), which may affect from individual physiological responses to the distribution and community structure of plants (Chaturvedi and Shivaji, 2006; Rumpf et al., 2018; López-Angulo et al., 2019; Sklenář et al., 2021). Such environmental heterogeneity has been studied mainly along altitudinal gradients and contrasting slope exposures. Specifically, soils of pole-facing slopes tend to be wetter, colder and with higher organic matter than those of equatorial-facing slopes (Scherrer and Körner, 2011). These differences in environmental conditions propitiate greater plant species richness and cover in polar than equatorial facing slopes (Zeng et al., 2014; Måren et al., 2015; Viale et al., 2019). While the difference in plant diversity of high-elevation species between slopes has been widely studied (e.g., Zeng et al., 2014; Yang et al., 2020), differences for other organisms such as PGPBs have been almost unexplored. To date, bacterial diversity decreases with altitude (Adamczyk et al., 2019). Meanwhile, their relative abundance, especially in free-living psychrophiles, increases with elevation (Siles and Margesin, 2016; Rui et al., 2022). PGPBs natives to alpine systems are essential nowadays due to their ability to cope with low temperatures and to promote plant growth through different mechanisms under cold conditions and poor nutrient soils (Chaturvedi and Shivaji, 2006; Jaggi et al., 2020; Farooq et al., 2022). These constraint conditions for plants can be alleviated by PGPBs through the activity of enzymes and phytohormones that favor plant nutrient uptake and stress resistance (Haselwandter et al., 1983; Widawati and Suliasih, 2006; Joshi et al., 2014; Yarzábal, 2014; Kadioglu et al., 2018; Pandey and Yarzábal, 2019; Fan et al., 2021). Some studies have reported PGPBs in alpine ecosystems (Yadav et al., 2015), described their diversity in terms of factors structuring bacterial communities (Tang et al., 2020; Wang et al., 2020; Rui et al., 2022), and quantified their plant growth-promoting activity (Haselwandter et al., 1983; Widawati and Suliasih, 2006; Viruel et al., 2011; Yadav et al., 2015; Kadioglu et al., 2018). Although the interaction with beneficial microorganisms could be part of the adaptive strategy of plants in alpine systems (Farooq et al., 2022), little is known about their abundance and activity of PGPB in contrasting microhabitats within these natural systems.

The Nevados de Chillán volcanic complex is in a Mediterranean – Temperate climate transition zone (Arroyo et al., 2004), characterized by a complex topography (González-Ferrán, 1995; Dixon et al., 1999), and great plant diversity and endemism (Rodríguez et al., 2008). The lower portion of the alpine zone is dominated by xerophytic vegetation, which is one of the southernmost xerophytic formations in Chile (Squeo et al., 2008). Plant species of this Andean xerophytic formation must cope with low soil moisture and large-daily thermal oscillations during the snow-free period in the area, which could be even more extreme depending on slope exposure. The success of xerophytic plant species in alpine systems could be mediated by PGPBs-plant associations. However, knowledge is scarce, with only one study reporting PGPB isolated from the shrub species *Parastrephia quadrangularis* in the Andean Puna (Acuña et al., 2019). Thus, our general goal was to study the abundance and activity of different plant growth-promoting bacteria (PGPB) associated with two abundant plant species of Andean xerophytic formations on contrasting slopes. We expected that the abundance and activity of different PGPB associated with plant species will be greater on drier and or more thermally extreme slope.

## Materials and methods

**Figure 1:**
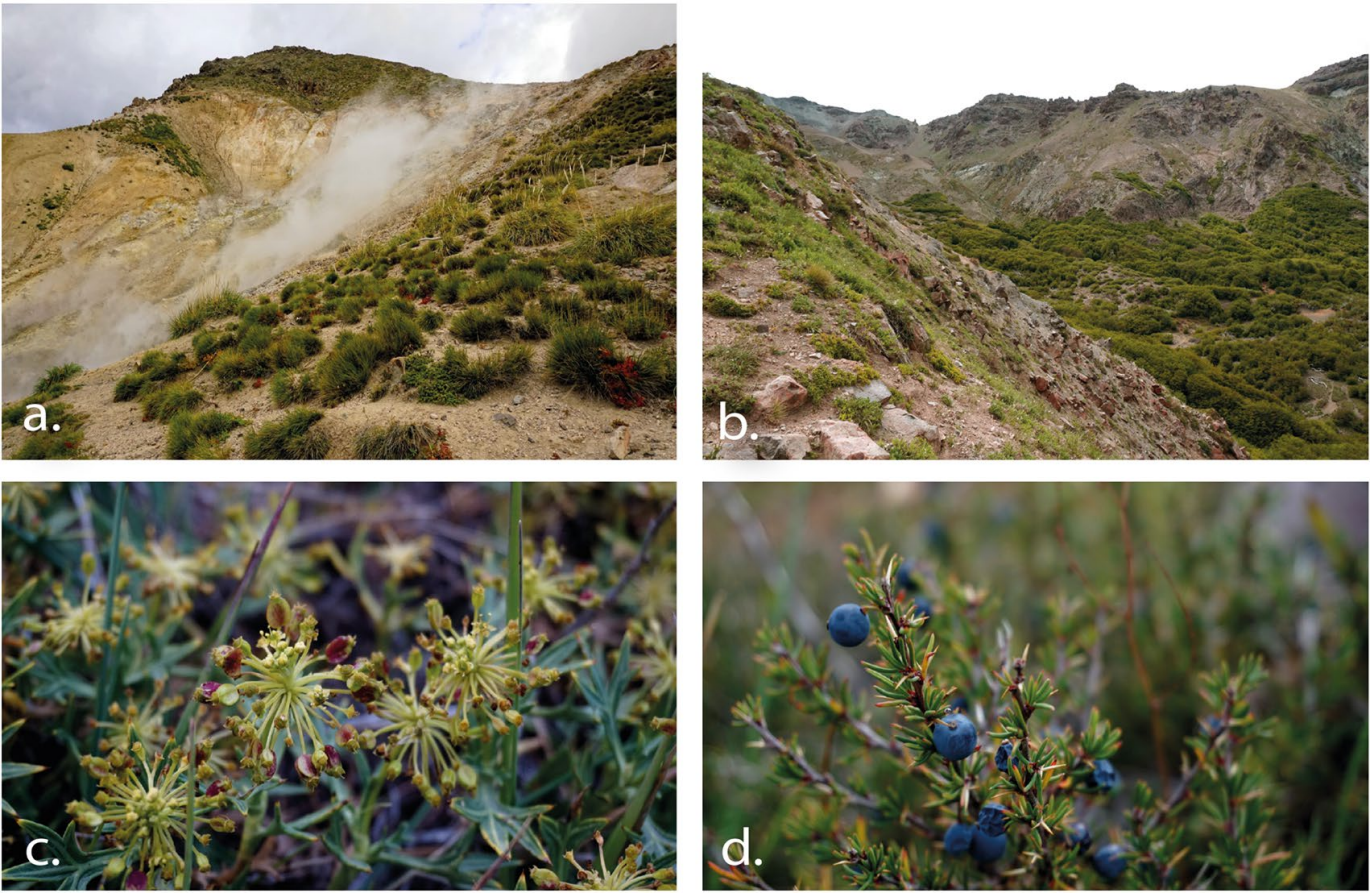
Study site located in the Nevados de Chillán Volcanic complex. Letters **(A,B)** indicate the north and south slopes, respectively. Selected species present on both slopes are indicated on the right side: **(C)**. *A. prolifera* and **(D)**. *B. empetrifolia.* Credits: Carla Aguilera-Torres.

**Figure 2:**
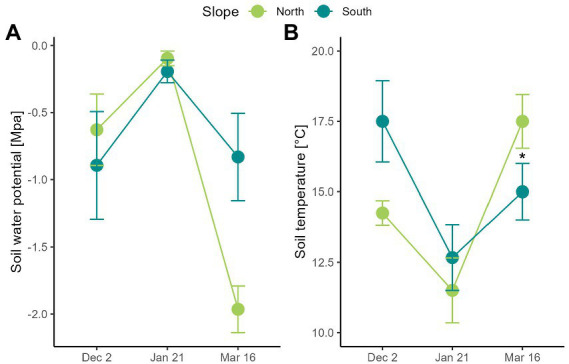
Soil climatic conditions measured in two contrasting slopes in the Nevados de Chillán Volcano complex. **(A)** Water potential (MPa) and **(B)** soil temperature (°C) was measured at 15 cm of soil depth on two contrasting slopes. Values correspond to average ± SE (*n* = 3). Dashed grey lines indicate temperature thresholds for plant physiology: heat (30°C) – freezing (0°C). Blue arrows indicate snowfall. Asterisks show significant differences (Repeated measures ANOVA, *p* < 0.05).

The study area was the Fumarolas sector (36°55′15”S, 71°26′25”W), nearby Nevados de Chillán Ski Resort, located at 80 km east of Chillán (Ñuble region, Chile). Is located in the transition zone between central Chile’s Mediterranean regions, with sclerophyllous and temperate forests plant elements of South Chile (Arroyo et al., 2004). This area is characterized by a past and current volcanic activity resulting in a manifold topography characterized by a complex relief and geology (González-Ferrán, 1995; Dixon et al., 1999). The combination of biogeographic situation, geomorphologic complexity, and climate change leads to an exceptional degree of botanical diversity and endemism (Rodríguez et al., 2008). In this area, the treeline is determined by *Nothofagus pumilio* and *N. antarctica* at approximately 2,100 m above sea level (Fajardo et al., 2011; Piper et al., 2016). Above there, the alpine zone comprises an elevation range from 2,100 to 2,700 m (Pfanzelt et al., 2008). Vegetation is short (<150 cm), and arranged in plant assemblages of shrubs, rosettes, grasses, and geophytes (Pfanzelt et al., 2008). For example, lower elevation portion (2100–2,300 m) comprises several shrubs (*Berberis empetrifolia*, *Azorella prolifera*) and herbaceous species (*Adesmia emarginata, Grausa lateritia*) assemblages, which coincides with one of the southmost xerophytic formations in Chile (Figure 1; Donoso, 1982; Squeo et al., 2001; Moreira and Cereceda, 2013). Upper elevation portion (2300–2,700 m) is dominated by herbaceous species (*Viola volcanica*, *Nassauvia revoluta*) and grasses (*Bromus, Festuca,* and *Poa* species), which forms a matrix of low plant cover between rocks and lava flows (Pfanzelt et al., 2008). In general, this Andean system is characterized by summer water shortage and great daily temperature fluctuations (Termas de Chillán climatic station, CN360042, 1708 m above sea level).^1^ During the snow free period, i.e., between October and April approximately, daily minimum and maximum air temperatures oscillate between −10°C to more than 41°C, respectively (Table 1), depending on slope and date. Moreover, soil water potential decrease from −0.8 MPa in October to <−2 MPa in March, especially in north-facing slopes (Figure 2).

**Table 1:** Air temperature and moisture conditions measured on two contrasting slopes in the alpine zone of the Nevados de Chillán volcano complex.

**Variable**	**North-facing slope**	**South-facing slope**
Snow free period (d)	174	140
Mean temperature (°C)	11.9 ± 0.1^a^	12.3 ± 0.1^a^
GDD_0_ (°C day^−1^)	2363.4 ± 153^a^	1846.8 ± 78.5^b^
Thermal breadth (°C)	22.1 ± 0.3^a^	18.2 ± 0.3^b^
Minimum temperature (°C)	2.6 ± 0.2^a^	4.3 ± 0.2^b^
Freezing intensity (°C)	−2.5 ± 0.2^a^ [−9.9]*	−2.3 ± 0.3^a^ [−6.6]*
Freezing duration (h)	4.1 ± 0.3^a^	5.5 ± 0.6^b^
Freezing frequency (%)	29.3^a^	11.4^b^
Maximum temperature (°C)	24.6 ± 0.4^a^	22.5 ± 0.6^b^
Heat intensity (°C)	33.5 ± 0.3^a^ [41.3]*	32.2 ± 0.2^b^ [35.8]*
Heat duration (°C)	3.2 ± 0.2^a^	2.4 ± 0.3^b^
Heat frequency (%)	48.9^a^	27.9^b^
Mean Relative Humidity (%)	51.9 ± 0.2^a^	51 ± 0.3^a^
Min Relative Humidity (%)	21.9 ± 0.6^a^	28.4 ± 1.2^b^
Max Relative Humidity (%)	84 ± 0.6^a^	80.4 ± 1.3^b^

*On the north-facing slope, freezing occurred on November 4, 2021, while heat occurred on January 17, 2022; on the south-facing slope, heat occurred on February 8 and freezing occurred on April 3, 2022. Values correspond to average ± SE (*n* = 3) for the entire snow free period between October 28, 2021, and April 19, 2022. Different upper cases indicate significant differences (*p* < 0.05). Extreme temperatures are shown between brackets as absolute values.

### Species selection

A pre-selection of six target species was conducted, based on Herbarium data at the Universidad de Concepción (CONC, Chile). This information was crossed with the national distribution of plant xerophytic formation report elaborated by National Forestry Service, CONAF [Gestión de Recursos Naturales (GRN), 2022]. The combined information added to field observations enabled to select within the study area two dominant species in the Andean xerophytic formations: *Berberis empetrifolia* Lam. (Berberidaceae) and *Azorella prolifera* (Cav.) G.M. Plunkett & A.N. Nicolas (Apiaceae; Figure 1). *B. empetrifolia* is a spiny and dwarf shrub of 50 cm tall, that has small narrow entire leaves, and small, yellow-colored flowers and later freshy blue-black berries. It is distributed from 29° to 56° S along Chile and Argentina, and from sea level to 3,500 m elevation (Rodríguez et al., 2018). *A. prolifera* is a shrub or subshrub that is native to Argentina, Bolivia and Chile, that forms cushions up to 100 cm high, yellowish-green, sometimes glaucous, very aromatic, petiolate leaves measuring up to 50 mm long, with yellow flowers, hermaphrodite in the center and male at the edge, arranged in simple umbels protruding above the foliage, its fruit is a winged reddish-yellow schizocarp. *A. prolifera* is distributed from 26° to 56° S, and from o to above 1,500 m of elevation (Rodríguez et al., 2018). Both species are key for other plants recruitment in the southern Andes (Nuñez et al., 1999) and exhibit high resistance to different types of stress (Durante et al., 2011; Golluscio et al., 2011; Varas et al., 2013; Sierra-Almeida et al., 2016).

### Microclimatic characterization of contrasting slopes

Target species were present in two contrasting slopes (Figure 1): north- (1,984 m, 36°54′24.25”S 71°24′10.16”W) and south-exposure (2,089 m, 36°54′16.90”S 71°23′58.83”W). On each slope, three air temperature (°C) and relative humidity (%) sensors were placed at 15 cm above the soil surface (U23 Hobo Pro v2 6′, Onset Comp, United States). They were programmed to record every 30 min during the entire snow free period. On the north-facing slope, sensors were installed on October 28, 2021, whilst on the south slope, they were installed on December 02 because it was covered by snow before this date. Air temperature recording were used to estimate several parameters: seasonal mean, maximum and minimum air temperatures; intensity, frequency, and duration of freezing (T < 0°C) and heat events (T > 30°C); growing degree days (GDDs), used as a measure of heat accumulation (in °C) above a base temperature (i.e., 0°C) to represent an index of the cumulate energy available to growing plants (McMaster and Wilhelm, 1997). It was calculated as:

GDD_0_ = [[(maximum daily temperature + minimum daily temperature)/2]] − base temperature.

The daily GDDs were summed per the entire growing season on each slope. We used 0°C as a conservative base growing temperature (threshold temperature above which plants can perform metabolic functions), because plants from cold climate generally vary in their absolute base growing temperature, and this value encompasses this variability (Körner, 2011).

Soil temperature and water potential were recorded by using psychrometers (PST-55, C52 Wescor Inc., Utah, United States), which were placed at 15 cm of soil depth (*n* = 3 per slope). These sensors were buried at the same date as air sensors. Soil temperature and moisture were manually recorded once per month using a microvoltmeter (HR 33 T; Wescor Inc., Utah, United States). In April, measurements of soil temperature and water potential were not carried out because started snowfall. All sensors were removed from the sites on April 19, 2022.

Given that soil chemical properties affect the bacterial abundance and activity (Preem et al., 2012; Chodak et al., 2015), on April 16, soil samples were collected under the plant canopy for their chemical characterization. A total of 12 soil samples of 1 kg (3 replicates × 2 species × 2 slopes) were placed into plastic bags, marked, and immediately transported into a cooler to our lab group at the University of Concepción (Concepción). Then, soil samples were sent for complete chemical analysis to the Soil and Plant Analysis Laboratory at Universidad de Concepción (Chillán).

### Bacterial abundance

On April 19, soil samples were collected from surrounding roots of *Azorella prolifera* and *Berberis empetrifolia.* Three soil samples of 0,5 kg were collected per species and slope (total 12 samples). Samples soils were placed into a cooler and immediately transported to our lab, where they were stored at −20°C until the start of microbiological studies. Abundance of bacteria were estimated by Colony-Forming Unit (CFU) per species (*A. prolifera* and *B. empetrifolia*) and slope (North and South). For this, 1 g of soil per species and slope exposure was taken and serially diluted in Phosphate-Buffered Saline (PBS) medium under sterile conditions. Subsequently, the 10^−2^ and 10^−3^ dilutions were sown on plates with plate count agar medium (DIFCO), which were incubated for 2 days at 25°C. The CFU of each dilution was then counted to determine the most abundant and morphologically different culturable strains for plant growth-promoting trait assays. The CFU were transformed to their natural logarithm (Log CFU g^−1^).

### PGPB activity

The five most abundant and distinct colonies by species and slope were selected according to their bacterial cell shape and structural appearance. They were subculture two or three times until pure strains were obtained. Nineteen culturable bacterial strains were obtained and subjected to assays that evaluated the activity of four plant growth-mechanisms, including IAA production, ACC deaminase, phosphate solubilization and nitrogenase enzyme activities and then were compared between contrasting slopes.

- To evaluate **indole acetic acid (IAA) production**, selected culturable strains were inoculated in generic nutrient broth [1 gD (+)-glucose, 15 g peptone, 6 g NaCl, 3 g yeast extract], with 0.15% (w/v) tryptophan in darkness for 96 h at 30°C with 120 rpm. IAA production was measured *via* high-performance liquid chromatography (HPLC; Primaide, Hitachi Co, Ltd., Tokyo, Japan; Gang et al., 2019). The calibration curve was prepared with serial solutions of IAA ranging from 0 to 50 ppm in methanol, 50 μl of the samples were injected onto a kromasil C-18 column equipped with a diode array detector, and retention times ranged from 5.7 to 7.9 min. All samples were analyzed in triplicate. The results were expressed in μg ml^−1^ of bacterial IAA.
- The presence and activity of **ACC-deaminase enzyme** was studied following protocols described by Senthilkumar et al. (2021) and Penrose and Glick (2003) with modifications. Liquid DF minimal culture medium was prepared, where the bacteria of interest were inoculated at 28°C for 72 h; bacteria that showed turbidity were seeded in solid DF minimal medium to confirm the presence of ACC deaminase. In cultures that evidenced plate growth in solid DF medium, ACC deaminase enzymatic activity was measured by the production of α-ketobutyrate, which was determined by spectrophotometer (Spectroquant Prove 300) at 540 nm by comparison with the standard curve of α-ketobutyrate, which ranged from 0 to 100 μM (Honma and Shimomura, 1978). Higher concentrations of α-ketobutyrate were indicative of higher enzyme activity (Ali et al., 2014).
- **To determine nitrogenase enzyme activity**, strains were incubated in a semi-solid nitrogen-free (NF) medium for 72 h at 28°C (Kifle and Laing, 2015). Samples showing evidence of turbidity were subjected to acetylene reduction assay (ARA; Hardy et al., 1968), where 10% of the atmosphere was removed with a syringe and replaced with acetylene. After 20 h, 4.4 ml of gas was taken for each vial to analyze the amount of ethylene formed through gas chromatography (GC 6890 N, Agilent Technologies), equipped with J&W HP-5 GC column, 30 m, 0.25 mm, 0.25 μm, H₂, N₂, and air detectors, with flow rates of 40, 24, and 450 ml/min, respectively. To determine the injection, detection and column temperatures, the ranges of Hara et al. (2009) were followed with modifications: Injection temperatures were 150°C, detection temperature was 150°C, and column temperature was 50°C. Retention times ranged from 1 to 1.5 min. 4–5 ml of samples were injected per 10 min. Each sample was prepared in triplicate for injection. The calibration curve was prepared with solutions composed of different ethylene concentrations expressed in nmol C₂H₄ d^−1^vial^−1^.
- **Phosphate solubilization,** mineral phosphate solubilization was assayed according to Pikovskaya (1948), the mineral phosphate solubilization was assayed by seeding bacteria in a solid medium Pikovskaya (PVK) in plates containing insoluble tricalcium phosphate, that bacteria with phosphatase are capable of degrading. The plates were incubated at 28°C. The development of a clear zone around the colony was evaluated after 20 days. Samples were analyzed in triplicate.

### Identification of bacterial strains by studying the 16S rRNA gene

To determine whether the selection of bacterial strains by cellular and structural appearance included different species, the following analysis were carried out. Ten bacterial strains randomly selected of 19 used for PGPB analysis were grown on semi-solid TBS medium. DNA extractions of individual colonies were performed using the DNeasy^®^ Plant mini kit (QIAGEN, Dusseldorf, Germany) according to the manufacturer’s instructions. The integrity of the DNA samples was visualized by agarose gel electrophoresis, the concentration was determined by spectrophotometry (A260/A280) using Infinite^®^ M200 Pro NanoQuant (Tecan^®^, Tecan Trading AG, Switzerland) and the DNA samples were kept at −20°C. PCR was performed to amplify and sequence part of the 16S rRNA gene. PCR reactions contained 1X GoTaq^®^ reaction buffer (1.5 mM MgCl_2_), 200 mM dNTP, 0 2 μM of each primer (16S_27F, 5’-AGAGAGTTTGATCCTGCTCAG-3′ and 16S_805R, 5’-GACTACHVGGGGGTATCTAATCC-3′), 1 U of GoTaq DNA Polymerase (Promega, Madison, United States), 20 ng/μL^−1^ of DNA and filled to 20 μl with sterile filtered molecular grade water. Thermal conditions were achieved by cycling at 94°C for 3 min, followed by 35 cycles of DNA denaturation at 94°C for 60 s, primer annealing at 45°C for 40 s and DNA elongation at 72°C for 60 s, and a final extension at 72°C for 7 min. PCR products were run on 1% (w/v) agarose gels with SYBR Safe DNA Gel Stain (Thermo Fisher Scientific, Inc., Carlsbad, CA) in TAE buffer at 80 V for 45 min. The amplicons were visualized in a UV light transilluminator. The amplified DNA fragments were purified and directly sequenced in both directions (Macrogen, Seoul, South Korea). Sequencing results (ABI chromatograms) were analyzed in the free open-source software UGENE v.33. Nine out of 10 bacterial strains yielded analyzable chromatograms. The consensus fastq files were analyzed using the EzBioCloud 16S Database (Yoon et al., 2017). EzBiocloud is a species level resolution database made of 61 700 species/phylotypes, including 13 132 species/phylotypes with validly published names. A phylogenetic tree was constructed using the Seaview 4.0 program with the neighbor-joining method to determine relationships between bacterial strains. The resulting consensus tree was constructed using 1000 replicates.

### Data analysis

Differences in soil conditions (i.e., temperature and water potential) throughout the growing season between north and south slopes were assessed by repeated measures ANOVAs (*p* < 0.05). Differences in mean, maximum, minimum air temperature and relative humidity, thermal breadth, intensity and duration of freezing and heat events between north and south-facing slopes, were assessed by *t* tests or non-parametric equivalent tests (*p* < 0.05) when data normality and homoscedasticity were not met. Differences in frequency of freezing and heat events and GDDs between slopes were assessed by Chi^2^ tests. Differences in soil properties were assessed by Factorial ANOVAs, where species and slope were predictors. When parametric requirements were not met these differences were assessed by Kruskal-Wallis ANOVA by ranks. Differences in PGPB abundance (Log CFUg^−1^) and in the activity of plant growth-promoting mechanisms (i.e., phytohormone and enzymes) were assessed by ANOVAs as well. All data were analyzed through the Statistica version 13.

## Results

### Microclimatic characterization of contrasting slopes

The North-facing slope was potentially more stressful than south-facing slope according to microclimatic data obtained during the complete snow free period (Table 1). Although air mean temperature was similar between slopes (*t* = 0.02, *p* = 0.985), GDD_0_ were 22% (χ^2^ = 3,133, *p* < 0.0001) and thermal breadth was 3.9°C greater (*t* = 7.3, *p* < 0.001) in north- than in south-facing slope. In addition, considerable differences in extreme temperatures were observed (Table 1). For example, the mean minimum temperature was lower in north- than in south-facing slope (2.5°C ± 0.2 vs. 4.3°C ± 0.2, *t* = 4.2, *p* < 0.0001). The intensity of freezing events averaged −2.4°C for both slopes (*t* = 0.11, *p* = 0.74), but their frequency was 2.4 times greater in north- than in south-facing slope (χ^2^ = 10.7, *p* = 0.001), with the lowest temperature of −9.9°C recorded on November 4, whilst south-facing slope was covered by snow. The duration of freezing events was 25% longer in south- than north-facing slope (*t* = 5, *p* = 0.028), but in both microsites they lasted on average more than 4 h (Table 1). At the other extreme, the mean maximum air temperature was 2.2°C higher in north- than in south-facing slope (Table 1, *Z* = 2.86, *p* = 0.004). In north-facing slope, heat events were 1.8 times more frequent (χ^2^ = 9.8, *p* = 0.002), 1.3 times longer (*Z* = 2.3, *p* = 0.022) and 1.3°C hotter (*Z* = 2.5, *p* = 0.011) than in the south-facing slope. In addition, heat events with temperatures over 40°C were recorded only in the north-facing slope (Table 1). Regarding air relative humidity (RH), averaged 51% for both slopes (*t* = 2.7, *p* = 0.077), but minimum RH was 6.5% lower (*t* = 3.9, *p* = 0.001) and maximum RH was 4% higher (*t* = 2.1, *p* = 0.034) in north- than in south-facing slopes.

Soil temperature was similar on north and the south-facing slope (Figure 2; slope F_1,4_ = 0.5, *p* = 0.519) with variations reported throughout the growing season (*date* F_2,8_ = 15.3, *p* = 0.002). In contrast, soil water potential was on average − 0.7 MPa and − 0.5 MPa in December and January on both slopes, respectively (Figure 2). However, its decrease was 2.2 times greater in the north- than in the south-facing slope (*date x slope* F_2,8_ = 9.3, *p* = 0.008). Hence, soils of north-facing slopes reported water potential of −1.96 MPa in March, while on the south-facing slope it was of −0.89 MPa (Figure 2). No recordings were carried out in April because snow started to cover soil moisture sensors.

Soil chemical properties varied between plant species and slope exposure (Table 2). For example, soil pH under *Azorella prolifera* was on average greater than of under *Berberis empetrifolia* (6.3 *vs* 5.3, H_3,12_ = 8.44, *p* < 0.038), independent of slope. Regarding organic matter, slope differences were observed for soils under both species but in opposite directions (Table 2; H_3,12_ = 9.15, *p* < 0.027). Under *A. prolifera* canopy, organic matter was a half lower in the north than in the south slope (Z = 1.96, *p* = 0.049). In contrast, organic matter was almost once greater in the north than in the south slope (Z = 1.96, *p* = 0.049) in soil collected under *B. empetrifolia* canopy (Table 2). Available K was 3 times greater in south than north slope for soils under *A. prolifera* (Z = 1.96, *p* = 0.049), whilst for soils under *B. empetrifolia* available K was similar between slopes (Z = 1.09, *p* = 0.275). Exchange Al was similar between slopes in soils under *A. prolifera* (Z = 0.86, *p* = 0.383), but it was almost 3 times greater in soils from north than south slopes under *B. empetrifolia* (Z = 1.96, *p* = 0.049). Al and Ca saturation varied between species but were similar between slopes (Table 2). For example, Al saturation was on average 15 times greater in soils under *B. empetrifolia* than in soils under *A. prolifera* (H_3,12_ = 9.26, *p* < 0.026). In contrast, Ca saturation was on average 26,4% greater in soils under *A. prolifera* than under *B. empetrifolia* (Table 2, F_1,8_ = 66.8, *p* < 0.0001). In the case of K Saturation, it was greater in soils from south slope independent of plant species (Table 2; H_3,12_ = 9.36, *p* < 0.025), with slope differences of 2.6 and 3.7 times in soils under *A. prolifera* and *B. empetrifolia*. Mg saturation exhibited similar percentages between slopes for soils under *A. prolifera*, but it was 8.2% greater in north than in south slope in soils under *B. empetrifolia* (Table 2; F_1,8_ = 10.9, *p* = 0.011). Similar pattern was observed in Fe (Table 2). No differences in Fe were found between slopes for soils under *A. prolifera*, but Fe was 44.2% greater in north than in south slope in soils under *B. empetrifolia* (Table 2; H _1,6_ = 3.86, *p* = 0.049). Regarding S available, it was almost 9 times greater in south than in north slopes in soils under *A. prolifera*, but no differences between slopes were observed in soils under *B. empetrifolia* (Table 2; F_1,8_ = 12.9, *p* = 0.007).

**Table 2:** Chemical characterization of the soils collected under plant canopy of *Azorella prolifera* and *Berberis empetrifolia* species growing in contrasting slopes.

**Chemical properties of the soil**	***Azorella prolifera***		***Berberis empetrifolia***	
	North-facing	South-facing	North-facing	South-facing
pH (water)	6.4 ± 0.1^a^	6.3 ± 0.3^a^	5.3 ± 0.1^b^	5.3 ± 0.2^b^
Organic Matter (%)	0.9 ± 0.2^a^	1.9 ± 0.1^bc^	2.5 ± 0.2^b^	1.6 ± 0.1^c^
N-NO_3_ (mg kg^−1^)	2.8 ± 0.4^a^	2.3 ± 1.5^a^	4 ± 1.3^a^	8.4 ± 2.2^a^
N-NH_4_ (mg kg^−1^)	1.8 ± 0.4^a^	1.3 ± 0.2^a^	2.7 ± 0.3^a^	1.7 ± 0.2^a^
Available Nitrogen (mg kg^−1^)	4.6 ± 0.1^a^	3.6 ± 1.6^a^	6.7 ± 1.3^a^	10.1 ± 2.4^a^
Olsen P (mg kg^−1^)	4.3 ± 1.7^a^	3.9 ± 0.5^a^	4.3 ± 1.1^a^	3.9 ± 0.2^a^
Available K (mg kg^−1^)	53.5 ± 20.9^a^	162 ± 3.6^b^	150 ± 25.8^bc^	97.9 ± 18.6^c^
Exchangeable K (cmol kg^−1^)	0.2 ± 0.03^a^	0.4 ± 0.01^a^	0.4 ± 0.1^a^	0.3 ± 0.1^a^
Exchangeable Ca (cmol kg^−1^)	14.6 ± 2.7^a^	12.2 ± 3.4^a^	9.5 ± 3.4^a^	1.2 ± 0.2^a^
Exchangeable Mg (cmol kg^−1^)	1.4 ± 0.4^a^	1.2 ± 0.5^a^	3± 1.3^a^	0.3 ± 0.05^a^
Exchangeable Na (cmol kg^−1^)	0.06 ± 0.02^a^	0.03 ± 0.01^a^	0.04 ± 0.003^a^	0.02 ± 0.003^a^
Sum of bases (cmol kg^−1^)	16.3 ± 2.8^a^	13.8 ± 3.7^a^	12.9 ± 4.8^a^	1.8 ± 0.3^a^
Exchange Al (cmol kg^−1^)	0.1 ± 0.1^ac^	0.08 ± 0.05^a^	1.4 ± 0.3^b^	0.5 ± 0.1^c^
CEC (cmol kg^−1^)	16.5 ± 2.7^a^	13.9 ± 3.6^a^	14.4 ± 5^a^	2.2 ± 0.4^a^
Al saturation (%)	1.1 ± 0.8^a^	1 ± 0.8^a^	10.9 ± 2.4^b^	20.9 ± 3.8^b^
K saturation (%)	1.4 ± 0.1^a^	3.6 ± 1.15^b^	3 ± 0.5^b^	11.2 ± 0.2^c^
Ca saturation (%)	88 ± 3.6^a^	85.8 ± 3.8^a^	66 ± 1.2^b^	55 ± 3.5^b^
Mg saturation (%)	9.1 ± 2.6^a^	9.3 ± 2.4^a^	19.7 ± 1.5^b^	11.5 ± 0.5^a^
Available S (mg kg^−1^)	1 ± 0.5^a^	8.8 ± 1.4^b^	1.9 ± 0.4^a^	4 ± 0.6^a^
Fe (mg kg^−1^)	8.2 ± 1.4^a^	15.3 ± 5.1^a^	18.3 ± 2.3^a^	10.2 ± 0.3^b^
Mn (mg kg^−1^)	1.8 ± 0.3^a^	3.5 ± 0.1^a^	2.6 ± 1.1^a^	1.5 ± 0.5^a^
Zn (mg kg^−1^)	0.2 ± 0.03^a^	0 ± 0^a^	0.1 ± 0^a^	0.2 ± 0.03^a^
Cu (mg kg^−1^)	0.6 ± 0.1^a^	1.3 ± 0.4^a^	0.7 ± 0.1^a^	0.4 ± 0^a^
B (mg kg^−1^)	0.1 ± 0.03^a^	0.2 ± 0.03^a^	0.1 ± 0^a^	0.1 ± 0.03^a^

Values correspond to average ± SE (*n* = 3). Different lower-case letters indicate significant differences (*p* < 0.05).

### Bacterial abundance and their identity from Andean xerophytic formations

**Figure 3:**
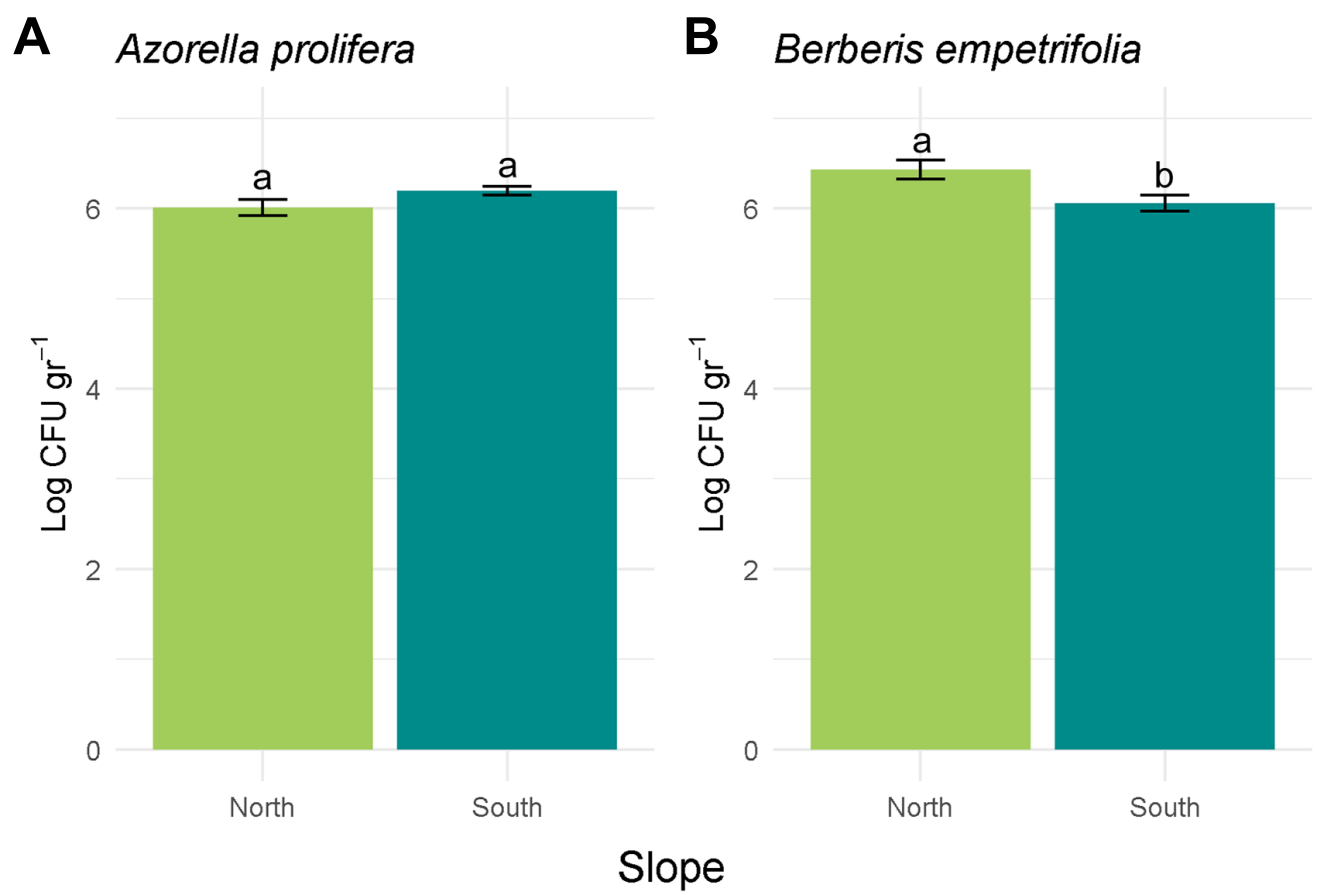
Bacterial abundance calculated in Log CFU g^−1^ for *A. prolifera* **(A)** and *B. empetrifolia* **(B)** soil. Values indicate average ± SE (*n* = 3). Letters indicate significant differences between slopes (Wilcoxon test, *p* > 0.05).

Differences in CFU abundance between contrasting slopes depended on plant species where soil samples were obtained (H_3,20_ = 7.91, *p* = 0.048; Figure 3). While abundance in soils collected under *Azorella prolifera* was similar between slopes (Figure 3A; Z = 1.43, *p* = 0.28), CFU were more abundant in soils from north-facing slope when they were collected under *Berberis empetrifolia* canopy (Figure 3B; Z = 0.57, *p* = 0.021).

### Slope differences in activity plant growth-promoting mechanisms

**Figure 4:**
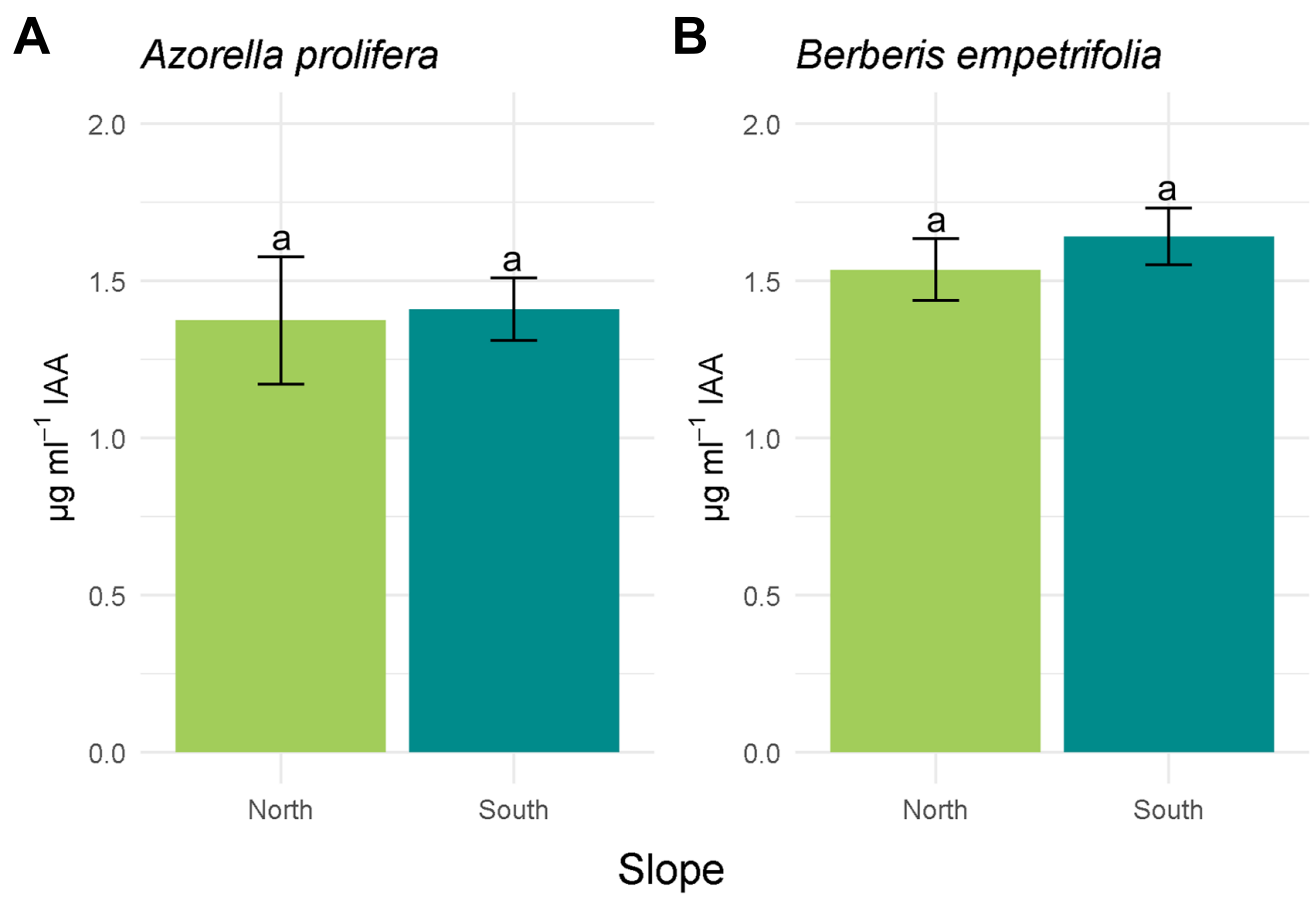
Indoleacetic acid (IAA) production of bacteria isolated from 2 species of Andean xerophytic formations on contrasting slopes: *Azorella prolifera* **(A)** and *Berberis empetrifolia* **(B)**. For each species, north slope is shown in green and south slope in light blue. Values indicate average ±SE. Equal letters indicate no differences (*p* > 0.05).

Of the 19 bacterial isolates present in the soils under the canopies of *A. prolifera* and *B. empetrifolia* on the north and south slopes, 89.47% of the isolates had activity in the production of IAA. Of the 17 isolates with activity in the indole acetic acid production mechanism, 52.95% of the isolates were from the northern slope and 47.05% were from the southern slope. For the ACC deaminase mechanism, 52.6% of the isolates had activity corresponding to 10 bacterial isolates, of which 70% corresponded to the northern slope and 30% to the southern slope. As for nitrogen fixation, 73.6%, corresponding to 14 bacterial isolates, had activity for this mechanism, where 64.28% corresponded to the northern slope and 35.73% had activity on the southern slope. 89.47% of the isolates corresponding to 17 bacterial isolates could solubilize phosphate, among the isolates with activity, we detected that 47.05% belonged to the northern slope and 52.95% (Supplementary Table S1; Supplementary material).The activity of four PGP mechanisms were compared between slopes. The hormonal PGP mechanism; Indole acetic acid (IAA) is presented in Figure 4. All isolates on the north slope of both *A. prolifera* and *B. empetrifolia* produced IAA, while on the south slope only 80% of the isolates produced this phytohormone. The highest value of IAA production was from an isolate from the north slope and *A. prolifera* soil (Supplementary Table S1; Supplementary material). When comparing the averages of IAA production on the north and south slopes for *A. prolifera* and *B. empetrifolia* species, we did not find differences between slopes, with averages IAA production of 1.4 μg ml^−1^ for *A. prolifera* (Figure 4A; F_1,22_ = 16.49, *p* = 0.9), and 1.6 μg ml^−1^ for *B. empetrifolia* (Figure 4B; F_1,25_ = 0,89, *p* = 0.46).

**Figure 5:**
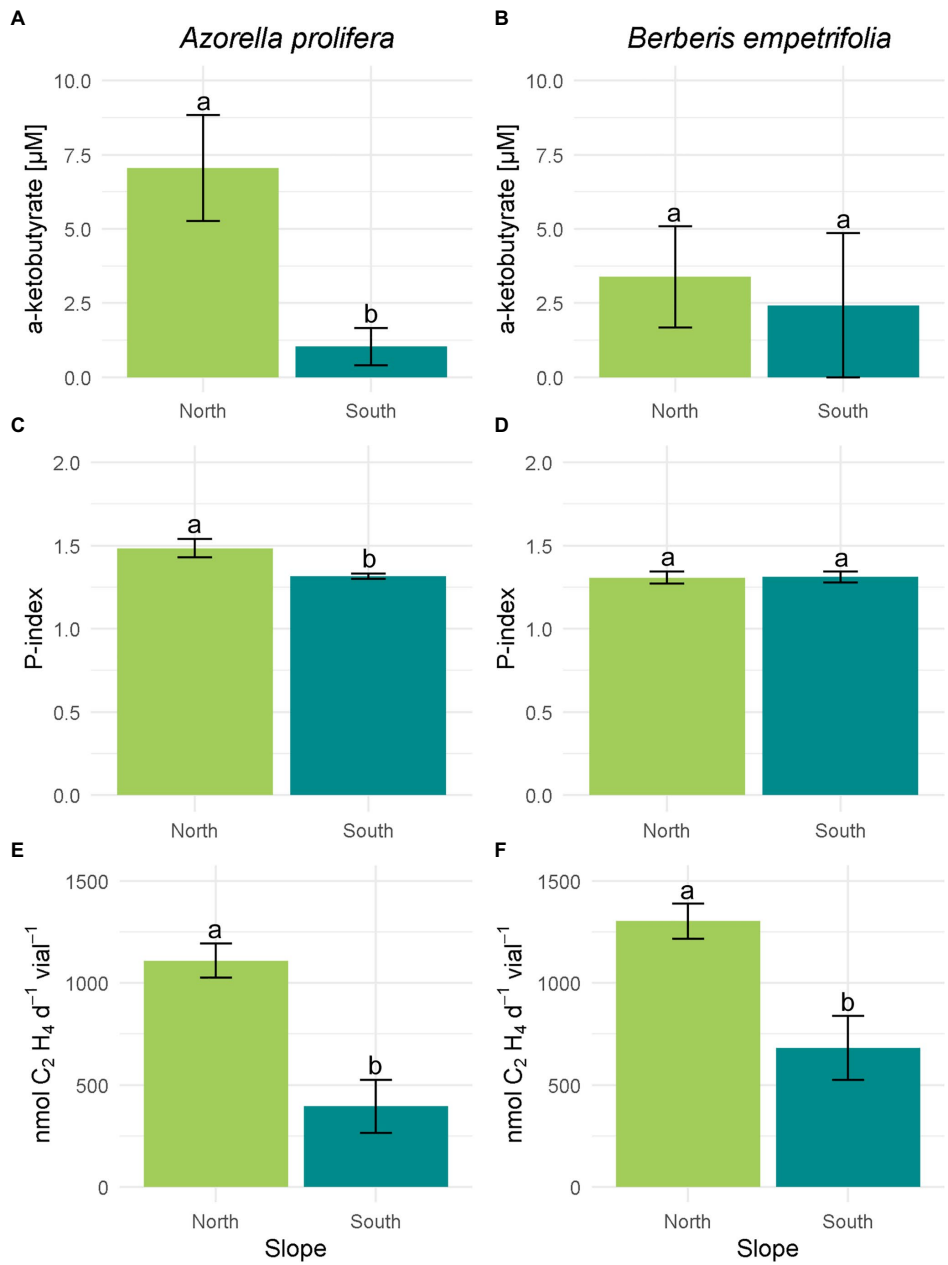
Enzymatic plant growth promoting mechanisms of bacteria isolated from 2 species of Andean xerophytic formations on contrasting slopes. For all mechanisms the columns were divided by species: *A. prolifera* on the left and *B. empetrifolia* on the rights. For each species the slopes are indicated with colors: green for the north slope and light blue for the south slope. ACC deaminase enzyme activity **(A,B)** are presented as μM of α-ketobutyrate. Phosphate solubilization **(C,D)** is presented as Index P. Nitrogen fixation **(E,F)** is given as nmoles C₂H₄ d^−1^ vial^−1^. Values indicate average ± SE. Different letters above error bars indicate significant differences between slopes (T test or nonparametric equivalent, *p* < 0.05).

The activity of enzymatic PGP mechanisms was on average higher in north than south slopes (Figure 5). On the north slope 77.7% of the bacterial isolates under the canopies of *A. prolifera* and *B. empetrifolia* possessed ACC-deaminase enzymatic mechanism activity, whereas, on the south slope, only 30% of the bacterial isolates from this slope possessed ACC-deaminase activity (Supplementary Table S1; Supplementary material). The highest activity of ACC deaminase was observed in a bacterial strain isolated from *A. prolifera,* which was 5 times higher in soils from north- than south slopes (7.1 vs. 1 μM; Figure 5A; F_1,7_ = 0.8 *p* = 0.01). In the case of *B. empetrifolia,* ACC deaminase had a similar activity in soils from both slopes (3.39 vs. 2.42 μM; Figure 5B; F_1,8_ = 0.04, *p* = 0. 41).

Among the north slope isolates, bacterial colonies with phosphate solubilizing mechanism were 88.8%, while on the south slope it was 90% (Supplementary Table S1; Supplementary material). However, the ability to solubilize phosphorus was higher on the north slope (H_3,20_ = 9.66, *p* = 0.022), particularly under the *A. prolifera* canopy, where, the solubilization Phosphorus index was 12% greater for culturable bacteria obtained in soils under from north than from south slope (Figure 5C; Z = 1.7, *p* = 0.01). In contrast, solubilization P index was similar between slopes for bacteria obtained from soil under *B. empetrifolia* (1,31 vs. 1,31; Figure 5D; Z = 0.21, *p* = 0.87).

Nitrogenase enzyme activity on the north slope occurred for all isolates, both for soils under *A. prolifera* canopy and *B. empetrifolia* soils. On the southern slope, on the other hand, nitrogenase activity only occurred for half of the bacterial isolates (Supplementary Table S1; Supplementary material). A greater nitrogenase activity was observed in bacteria obtained from north slope soils independent of plant species (Figures 5E,F). For example, for bacteria isolated from *A. prolifera* soils, ethylene production averaged 396 nmoles C₂H₄ d^−1^vial^−1^ on the south slope, whilst on the north slope it was 1,109 nmoles C₂H₄ d^−1^vial^−1^ (Figure 5E; F_1,25_ = 0.95, *p* = 0.007). For bacteria isolated from *B. empetrifolia* soils, the nitrogenase activity was 3.3 times greater in the north than in the south slope (Figure 5F; F_1,25_ = 3.5, *p* = 0.001).

### Molecular identification of bacterial strains

**Figure 6:**
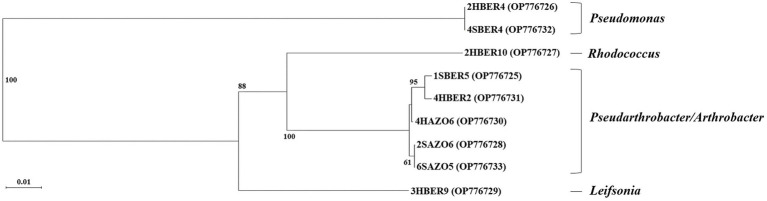
Phylogenetic tree based on sequences of the 16S genes (790 bp amplicon size) of 9 bacterial isolates obtained from *A. prolifera* and *B. empetrifolia* species from the north and south slopes of the Nevados de Chillán volcanic complex. 2HBER4, 2HBER10, 4HBER2, 3HBER9 correspond to isolates of the species *B. empetrifolia* on the south slope. 4SBER4, 1SBER5 correspond to isolates of the specie *B. empetrifolia* on the north slope. 4HAZO6 correspond to isolates of the specie *A. prolifera* on the south slope. 2SAZO6, and 6SAZO5 correspond to isolates of the species *A. prolifera* on north slope.

Randomly selected bacterial strains used to determine species identity of colonies and correspond to five genus and six species (Table 3). Regarding phylogenetic tree, four clades were identified and correspond with genus detected (Figure 6). Only two bacterial strains were assigned to the same species (2HBER4 and 4SBER4) and would correspond to *Pseudomonas atacamensis*. These bacterial strains where isolated from *B. empetrifolia*. Five bacterial strains were assigned to species belonging to the genera *Pseudarthrobacter* and *Arthrobacter*, detecting only almost identical (2SAZO6 and 6SAZO5) both from under *A. prolifera* on the south slope. The other two entities are made up of single species, respectively.

**Table 3:** Bacterial sequences obtained for 9 of the most abundant colonies isolated from soil under plant xerophytics species in the Nevados de Chillán Volcano Complex. See Materials and Methods for details.

ID	Plant species origin	Slope	Colony	Top-hit taxon in EzBioCloud 16S database	Similarity (%) in EzBioCloud 16S database	GenBank accession number
1SBER5	*Berberis empetrifolia*	South	5	Arthrobacter oryzae/A. pascens	99,37/99,21	OP776725
2HBER4	*Berberis empetrifolia*	North	4	Pseudomonas atacamensis	99,86	OP776726
2HBER10	*Berberis empetrifolia*	North	10	Rhodococcus qingshengii	99,44	OP776727
2SAZO6	*Azorella prolifera*	South	6	Arthrobacter globiformis/Pseudarthrobacter scleromae	99,53/99,52	OP776728
3HBER9	*Berberis empetrifolia*	North	9	Leifsonia soli	99,85	OP776729
4HAZO6	*Azorella prolifera*	North	6	Arthrobacter oryzae/A. pascens	99,85/99,85	OP776730
4HBER2	*Berberis empetrifolia*	North	2	Pseudarthrobacter sulfonivorans/Arthrobacter ginsengisoli/A. pascens	98,98/98,71/98,71	OP776731
4SBER4	*Berberis empetrifolia*	South	4	Pseudomonas atacamensis	99,85	OP776732
6SAZO5	*Azorella prolifera*	South	5	Arthrobacter oryzae/A. pascens	98,99/98.84	OP776733

## Discussion

This work quantified the abundance and activity of bacteria present in the south-central zone of the Andes Mountains. In addition, we observed how environmental variability in water availability, thermal oscillation, and plant identity impacted the different indicators evaluated. An important finding in the present investigation was that a higher abundance and enzyme activity was observed on the drier slope compared to the southern slope. In addition, two of the three enzymatic mechanisms showed differences between slopes, but only for bacteria associated with *Azorella prolifera*, not for *Berberis empetrifolia*. Thus, species identity is key when exploring the beneficial potential of plant growth-promoting bacteria in alpine systems.

First, the ranges obtained for bacterial abundance seem to coincide with the literature. For example, works such as those of Sinegani and Younessi (2017) have obtained ranges close to 3 Log CFU g^−1^ for contaminated sites, and values between 6.3 and 8.3 Log CFU g^−1^ for pastures and agricultural systems, the most common being close to 6 Log CFU g^−1^. Kieft et al. (1993) have obtained ranges between 2 and 6.7 Log CFU g^−1^ for soils of natural arid systems in the United States. In relation to the activity of PGPBs from Andean xerophytic formations reached low levels compared to those reported for agricultural systems. For example, our PGPBs produced indole acetic acid in a range of 1.4 μg ml^−1^ to 1.7 μg ml^−1^, whilst in agricultural systems IAA productions fluctuate between 8.5 μg ml^−1^ and 69.7 μg ml^−1^ (Herlina et al., 2017). Likewise, while phosphate solubilization in agricultural systems fluctuates between 4 and 5 (Schoebitz et al., 2013; Pande et al., 2017), in our Andean xerophytic system it did not exceed 1.5. While the highest nitrogenase enzyme activity in our assay was 1.4 μmol C₂H₄ d^−1^vial^−1^, assays with industrial bacteria ranged between 6 and 108 μmol C₂H₄ d^−1^ vial^−1^ (Li et al., 2017). Most studies evaluating the potential benefits of PGPBs are conducted in agricultural systems, with bacteria that have been optimized for years, so the low enzyme activity observed in bacteria from natural systems is not surprising. Interestingly, this pattern changes when we compare our results with the plant growth-promoting activity of bacteria from natural systems. For example, Kadioglu et al. (2018), isolated cold-tolerant bacteria with ACC deaminase activity from different native alpine plants in Erzurum (between 1,760 and 2,720 m above sea level). The activity of these bacteria ranged from 0.9 to 1.2 μM α-ketobutyrate, almost six times less than the activity obtained in our study. Most studies on the abundance and activity of PGPBs in alpine systems are generally from the Himalayas, where the bacteria interact with cultivated plants and in highly degraded soils (e.g., Majeed et al., 2018; Kumar et al., 2019). The level of disturbance in a system is also known to affect PGPB activity (Bergottini et al., 2015). This makes it difficult to draw comparisons that evidence the potential of the findings embodied in this study.

### Bacterial abundance and growth-promoting mechanisms related to slopes

We observed that bacterial abundance and enzyme activity was higher on the north than on the south slope. The climatic conditions could be behind this trend. Generally, the north slope tends to be drier and warmer than the south slope, as previously described for its equatorially oriented slopes (Scherrer and Körner, 2011). In addition, more intense and frequent heat and freezing events occurred on the north slope (Table 1), with greater intra-seasonal thermal fluctuations than on the south slope. This means that plant-PGPB interactions should be more likely to be found on the north slope, and the benefits, represented as the activity of PGP mechanisms, should be greater on this “hard” slope.

Regarding the influence of each environmental factor on bacterial abundance, what might contribute to the differences observed on the north slope. Studies have described the influence of each environmental factor independently, such as temperature (Chen et al., 2015), soil moisture (Naylor and Coleman-Derr, 2018), and soil chemical properties (Ai et al., 2020). For example, increasing temperature favors bacterial abundance, especially in cold regions (Chen et al., 2015). As for soil moisture, it affects in a more dynamic and integrated way, because it affects soil chemical properties and plant performance (Naylor and Coleman-Derr, 2018), which in turn affects bacteria. However, the identity and adaptations of bacteria determine whether they will survive and grow under extreme conditions (Pérez-Jaramillo et al., 2018). Furthermore, their response will depend on how stable the communities they compose are; stable bacterial communities do not change in drier soils (Stres et al., 2008). A clear example is a work of Chodak et al. (2015), who evaluated the response of drought-adapted bacteria that were subsequently rewetted, resulting in a stress-related decrease in gram-negative bacteria and an increase in gram-positive bacteria. Although the higher bacterial abundance observed on the north-facing slope responded to climatic factors, we believe that the response of bacterial abundance depends also on the characteristics of the bacteria (i.e., adaptations and identity), their community and the plants with which they interact.

The identity of the most abundant and active bacteria coincides with that reported for other systems with extreme conditions. All bacterial genera identified in this study have been identified in climatically limiting environments such as the Himalayas (e.g., *Pseudomonas*, Selvakumar et al., 2008; *Rhodococcus*, Ruberto et al., 2005; *Leifsonia*, Reddy et al., 2008), the Andes mountain range (e.g., *Pseudomonas* Viruel et al., 2011; Vega-Celedón et al., 2021), Antarctica (e.g., *Arthrobacter*, Dsouza et al., 2015 *Pseudarthrobacter*; Shin et al., 2020; *Leifsonia*, Ganzert et al., 2011), as well as in extremely dry regions (e.g., *Pseudoarthrobacter* isolated from African deserts, Buckley et al., 2019). In terms of their activity, the detected genera have been characterized as PGPB. For example, the production of IAA by bacteria of the genus *Leifsonia* (Kang et al., 2016), or the ACC deaminase, phosphatase, and nitrogenase activity of *Pseudomonas* (Georgieva et al., 2018; Adhikari et al., 2021; Vega-Celedón et al., 2021), or bacteria of the genera *Arthrobacter* and *Pseudoarthrobacter* that have been reported with multiple growth-promoting activities (Lahsini et al., 2022). It is believed that the climatic limitations of our study area favored a select group of PGPB that possess adaptations that allow them to survive and grow in conditions that for other bacterial groups would be lethal.

The influence of climatic variability concerning temperature and water availability on the enzyme activity of PGPB has been limitedly studied. For example, for ACC deaminase enzyme activity, we know that it is an enzyme characteristic of saline and drought-exposed soils (Glick, 2004; Brockett et al., 2012). In general, both the number of ACC deaminase isolates, and their activity were higher on the north slope than on the south slope, which was much less active. Considering our results, it could be intuited that in more extreme environments there could be a higher activity of this enzyme. The phosphatase activity has been detected in psychrotolerant bacteria, which also solubilize phosphate under low-temperature conditions [e.g., *Pseudomonas* spp. isolated from Hilamaya, Adhikari et al. (2021)]. Our studied bacteria likely possess the same qualities, but studies are lacking to understand the influence of temperature on phosphate solubilization of our isolates. About nitrogen fixation, which is also higher on the north slope, we know that nitrogenase enzyme activity is favored in humid and cold south conditions (Rousk et al., 2018). However, our results showed that nitrogenase enzyme activity was higher on the drier and more temperature fluctuating north slope. Probably, a specific group of nitrogen-fixing bacteria adapted to such microclimatic conditions prevails on the north slope. The exposure of the slope favored the activity of the three enzymatic mechanisms on the north slope. However, it was not enough to create differences between the two species, so we believe that plant identity played a key role in our results.

### Bacterial abundance and growth-promoting mechanisms related to species identity

Bacterial abundance was influenced by slope exposure, and unexpectedly by species identity. Initially, we thought that trends in abundance and activity should respond only to slope exposure and should coincide for the two species since both were present under the same temperature and moisture conditions. By looking at the chemical properties of the soil, there were noticeable differences between species. A clear example was the alteration of pH by plant identity, which exacerbated the differences in bacterial abundance only in *B. empetrifolia* soils. Soils of *B. empetrifolia* and *A. prolifera* had differences in pH, with the former being more acidic. Soil pH is usually altered by root exudates of plant identity (Youssef and Chino, 1989). However, studies that have evaluated bacterial abundance as a function of pH claim that increasing pH should increase bacterial abundance (Rousk et al., 2018). On the other hand, research such as that of Yang et al. (2019), conducted in alpine systems disturbed by agricultural activity, mentions that the abundance of groups such as Actinobacteria (e.g., *Rhodococcus* associated with *B. empetrifolia* on the North Slope) is not influenced by changes in pH (Yang et al., 2019). While we believe that the combination of slope exposure coupled with pH changes in *B. empetrifolia* soils benefited the bacterial abundance of a group of microorganisms, studies are lacking to secure these insights.

Linking plant identity and associated abundant bacteria. We found that for the genus *Azorella*, it is the first time that bacteria of the genus *Pseudoarthrobacter* are detected. Previously, works such as those of Rodríguez-Echeverría et al. (2021) had detected abundant phyla such as actinobacteria for *A. compacta* and *A. madreporica*. However, among the bacteria identified, no actinobacteria were reported for *A. prolifera*. Vega-Celedón et al. (2021) had previously detected bacteria of the genus *Arthrobacter* associated with *B. microphylla* in Patagonia. Genera such as *Pseudomonas*, *Rhodococcus*, and *Leifsonia* had not been previously detected, so this is the first record in this regard.

In this study, plant identity influenced two of the three evaluated mechanisms of PGPB. Interestingly, bacterial isolates present on the north slope under the *A. prolifera* plant canopies differed in activity for two of the four mechanisms evaluated between the north and south slopes. At the same time, these bacterial isolates were the only ones that presented activity in all the hormonal and enzymatic mechanisms evaluated (Supplementary Table S1; Supplementary material). Phosphatase activity was high in soils under the *A. prolifera* canopy, which in parallel exhibited the lowest percentage of organic matter on the north slope. Coincidentally, Alemneh et al. (2022) reported a negative correlation between phosphate solubilization and soil carbon, which explains our results. Concerning ACC deaminase enzyme activity, we believe that it is not linked to soil chemical properties and is related to the plant in a more complex way. We know that the plant through its root exudates modifies its microbiome benefiting one group of microorganisms over others for its benefit (Glick et al., 2007). *A. prolifera* was likely more stressed than *B. empetrifolia* on the north slope, and for this reason, we only had differences in ACC deaminase activity for one species. As for nitrogenase enzyme activity, this was higher on the north slope for both species, so we believe that at least this mechanism is not influenced by species identity. Our results evidence a notorious relationship between plants and PGPB activity, where interactions do not seem to occur randomly.

The combination of slope exposure and plant identity evidenced a clear influence on PGPB enzyme activity, where the harshness of the alpine system favored PGPB and its benefits when conditions became more extreme on the north slope. Studies indicate that stressed plants modify their root exudates, altering their soil microbiome and favoring the presence of PGPB (Li et al., 2020; Glick and Gamalero, 2021; Chen et al., 2022). The composition of root exudates depends on soil chemical properties, plant genotype, and plant age (Vives-Peris et al., 2018). Perhaps, climatically limiting conditions “enhanced” *A. prolifera* root exudation, increasing the activity of PGPBs. Further studies need to consider plant responses by incorporating root exudates among predictors of bacterial abundance and activity.

## Conclusion

Our work brings us closer to understanding what factors affect the abundance and activity of PGPB in hard systems such as those present in our system. Before our research, studies related to PGPB present in the soils of the Andes Mountains had evidenced their great biotechnological potential and their novelty for the detection of new species, in areas such as the Atacama Desert and the altiplano, with preliminary characterizations. The present work enriches the existing information related to the presence of PGPB from alpine systems in regions that had not been previously explored.

Our work showed how the environmental heterogeneity of the alpine systems, given by the contrasting slopes and its thermal and moisture oscillations, added to the identity of the species, generating variations in the abundance and plant growth promoting activity of the microorganisms present under the plant canopy of the xerophytic formations. It would be interesting to determine how and to what extent each factor contributes to the benefit provided by PGPBs. We believe that incorporating the influence of factors such as plant condition, plant identity, and microclimatic variations on changes in PGPB abundance and activity would highlight the importance of these interactions that do not occur randomly, especially when conditions become more extreme.

## Data availability statement

The original contributions presented in the study are included in the article/Supplementary material, further inquiries can be directed to the corresponding author.

## Author contributions

Plants may interact with soil bacteria that promote their survival and growth (PGPB) through a variety of mechanisms, especially under harsh climatic conditions. Plants that live in alpine systems have to be able to cope with harsh climatic conditions such as temperature extremes. Likely, plant-bacteria interactions are behind of plant strategies through they can grow in alpine systems. However, these interactions are poorly known. One of the southernmost xerophytic formations is in the Andes of central-southern Chile. In this system, plants are exposed to drought and extreme temperatures during the growing season, being harsher on equatorial than polar slopes. Thus, we compared the abundance and activity of PGPB under two plant species canopy that live in contrasting slopes. We found that bacterial abundance and enzymatic activity of PGPB was higher on the driest and with greatest thermal oscillation slope. Unexpectedly, we also found that abundance and activity of bacteria depended on plant identity. We believe that microclimatic conditions such as humidity, temperature, soil nutrients, and plant identity explain our findings, which are not random, especially when conditions become more extreme.

## Funding

This work was supported by CONAF-FIBN 047/2020 (RH), FONDECYT N°1220425 (MS), VRID-UDEC 2021000184INV (ASA) Grants, and ANID-Chile Technological Centres of Excellence with Basal Financing Project [CHIC–ANID PIA/BASAL PFB210018]. Carla Aguilera-Torres holds an ANID Master Fellowship.

## Conflict of interest

The authors declare that the research was conducted in the absence of any commercial or financial relationships that could be construed as a potential conflict of interest.

## Publisher’s note

All claims expressed in this article are solely those of the authors and do not necessarily represent those of their affiliated organizations, or those of the publisher, the editors and the reviewers. Any product that may be evaluated in this article, or claim that may be made by its manufacturer, is not guaranteed or endorsed by the publisher.

## Footnotes

^1^ https://climatologia.meteochile.gob.cl/ref
