# Supplementary material for: Relieving your stress: PGPB associated with Andean xerophytic plants are most abundant and active on the most extreme slopes
Source: Front Microbiol. 2023 Jan 18;13:1062414. doi: 10.3389/fmicb.2022.1062414 (PMC9889642; doi:10.3389/fmicb.2022.1062414)
Supplement: Supplementary file 1 [file Data_Sheet_1.PDF]

Supplementary Material

Table S1. Plant growth-promoting activity of different bacteria isolated under canopies of *Azorella prolifera* and *Berberis empetrifolia* plants on contrasting slopes. Values correspond to mean ± standard error.

Species	Slope	Colony	IAA [μg ml^{-1}]	ACC [μM α-ketobutyrate]	N [nmoles C_2H_4 d^{-1} $vial^{-1}$]	P [p index]
Azorella prolifera		2sazo_col11	1.8 ± 0.1	6.6 ± 0	1521.6 ± 43.5	1.4 ± 0.1
	North	2sazo_col6	2.2 ± 0	3.5 ± 0	1194.1 ± 23.8	1.8 ± 0.1
		4sazo_col5	0.8 ± 0	6.1 ± 0	925.6 ± 2.3	1.4 ± 0
		6sazo_col5	0.6 ± 0	12 ± 0	796 ± 3.8	1.5 ± 0
		1hazo_col20	1.7 ± 0	2.3 ± 0	1040 ±15.8	1.3 ± 0
		2hazo_col4	0.9 ± 0.1	2.8 ± 0	0	0
	South	3hazo_col5	1.6 ± 0.1	0	0	1.3 ± 0
		4hazo_col6	0	0	938.3 ± 5.5	0
		5hazo_col2	1.4 ± 0.1	0	0	0
Berberis empetrifolia		1sber_col3	1.2 ± 0.1	0	1005 ± 9.7	1.2 ± 0
		1sber_col5	1.9 ± 0.1	6.2 ± 0	906 ± 12.8	1.4 ± 0
	North	1sber_col6	1.1 ± 0.1	0	1455 ± 20.6	1.2 ± 0.1
		2sber_col1	1.7 ± 0	2.2 ± 0	1767.5 ± 123	1.4 ± 0
		4sber_col4	1.8 ± 0-1	8.5 ± 0	1377 ± 61.1	0
		2hber_col4	1.8 ± 0.1	0	1169 ± 84.3	0
		2hber_col10	1.2 ± 0.1	0	0	1.3 ± 0
	South	3hber_col9	1.9 ± 0.1	0	838.6 ± 11.3	1.4 ± 0.1
		4hber_col9	1.7 ± 0.1	12.1 ± 0	1400.4 ± 42.3	1.4 ± 0.1
		5hber_col2	0	0	0	1.3 ± 0.1
